# Magnitude and predisposing factors of intestinal parasitosis and tuberculosis coinfection at five health institutions in Southern Ethiopia: A cross‐sectional study

**DOI:** 10.1002/hsr2.1569

**Published:** 2023-09-20

**Authors:** Agumas Shibabaw, Mihret Tilahun, Alemu Gedefie, Zenawork Sahle, Melaku A. Belete, Hussen Ebrahim, Habtu Debash, Bekele Sharew

**Affiliations:** ^1^ Department of Medical Laboratory Sciences, College of Medicine and Health Sciences Wollo University Dessie Ethiopia; ^2^ Department of Medical Laboratory Sciences Debre Birhan Health Science College Debre Birhan Ethiopia

**Keywords:** coinfection, intestinal parasites, pulmonary tuberculosis, risk factors

## Abstract

**Background and Aims:**

Intestinal parasites affect the tuberculosis disease outcome by shifting the cell‐mediated to humoral immune response and host immune system suppression. However, *Mycobacterium tuberculosis* (MTB) infection favors the immune escape of parasites. Hence, exploring the rate of intestinal parasitic coinfection with pulmonary tuberculosis (PTB) and its predisposing factors to take better preventive, control, and management measures.

**Methods:**

A facility‐based cross‐sectional study was conducted from September to December 2020 at five health institutions in Hawassa city. A total of 214 PTB patients were diagnosed using the GeneXpert assay and enrolled in this study. Demographic, clinical, and risk factors data were collected using a structured questionnaire. Stool samples were collected using a clean, labeled, and leak‐proof stool cup. Stool samples were examined using direct saline microscopy and the formal‐ether concentration technique. The data were entered and coded in SPSS software for analysis. Bivariate and multivariate logistic regression were employed to identify the associated risk factors. A *p*‐value less than  0.05 was considered statistically significant.

**Results:**

The overall rate of intestinal parasitic‐MTB coinfection was 36.9%. The most dominant intestinal parasite was *Gardia lamblia* (17.8%, 38), followed by *Entamoeba histolytica*/*dispar* (9.3%, 20). Intestinal parasitosis coinfection of PTB was associated with being rural resident (adjusted odds ratio [AOR] = 2.42; 95% confidence interval [CI]: 1.23–4.8), not washing of fruits and vegetables before eating [AOR = 4.14; 95% CI: 1.92–9], being at the early stage of anti‐TB treatment [AOR = 3; 95% CI: 1.5–6.3] and presence of chronic diseases [AOR = 7; 95% CI: 3.4–14].

**Conclusion:**

The burden of intestinal parasites‐MTB coinfection was high. Those who wash fruits and vegetables before eating should be encouraged, early treatment of PTB patients and avoiding the practice of open‐field defecation, especially in rural communities, is necessary. The dual effect of coinfection on disease severity and treatment success needs further cohort study.

## 1 INTRODUCTION

Tuberculosis (TB) is a major cause of ill health.^1^ Until the current pandemic of severe acute respiratory syndrome coronavirus 2 (COVID‐19), TB was the leading cause of death. According to a World Health Organization (WHO) report, approximately 10 million people have TB and 1.5 million die in 2020. The COVID‐19 pandemic resulted in an increase in TB deaths due to reduced access to TB diagnosis and treatment.^1^ Ethiopia is a triple burden country, having a high proportion of TB/human immunodeficiency virus (HIV), TB, and multidrug‐resistant‐TB burden countries.^1^

*Mycobacterium tuberculosis* (MTB) infection prevention and control are mainly dependent on the strength and interaction of the innate and adaptive immune responses of the host. The TB disease outcome is mainly determined by cell‐mediated immunity.^2^, ^3^

In immunocompetent individuals, MTB infection is controlled by infected macrophages, CD4+ and CD8+ T cells, and the T‐helper (Th)‐1 response.^2^, ^4^ Those people whose immunity is suppressed and/or shifted to the Th‐2 response are more susceptible to acquiring TB infection. Malnutrition, helminths infection, diabetes mellitus, cancer, HIV, and administration of steroid drugs are the risk factors that suppress and/or shift the host immune response, and make people susceptible to TB infection.^2^, ^4^, ^5^

Intestinal parasites (IPs), specifically helminths, induce the Th‐2 response through cytokines production such as interleukin (IL)‐4, IL‐5, IL‐9, and IL‐13, and high levels of immunoglobulin E antibodies and eosinophils.^4^ These Th‐2 immune responses are controlled by the activation and expansion of both natural and inducible regulatory T cells.^5^, ^6^ All these immunomodulations favor the multiplication and dissemination of MTB for the development of active TB and granuloma formation.^7^, ^8^ These independent immunomodulations of helminths and MTB coinfection determine disease progression, pathogenesis, and outcome.^9^ IPs negatively affect the outcome of TB patients by changing the immune response from cell‐mediated to humoral, and leading to the host immune system suppression. On the other hand, TB infection creates a favorable environment for IPs.

The burden of parasitic infections is alarming in Sub‐Saharan Africa. Patients infected with parasitic infections are more susceptible to MTB infection due to the development of a dominant Th‐2 response.^2^, ^3^

Worldwide, approximately 3.5 billion and over 450 million people are affected by and ill with IPIs, respectively.^10^, ^11^ IPs and TB infections affect primarily the low socioeconomic status populations and overlap greatly in geographic distribution.^3^, ^11^, ^12^

Intestinal parasitic coinfections are the major public health problems in Ethiopia.^13^, ^14^, ^15^ A high rate of MTB‐IP coinfection was reported in Gondar, Arba Minch, and Addis Ababa, Ethiopia.^3^, ^16^, ^17^ A study conducted in Addis Ababa and Arba Minch revealed that 22% and 26% of intestinal helminths coinfection rates were reported, respectively.^16^, ^17^ This coinfection would increase the complexity of control and prevention of TB and parasitic diseases.^12^, ^13^, ^15^, ^16^, ^17^, ^18^ The efficacy of the Bacille Calmette Guerin vaccine is limited against TB epidemic areas in resource‐limited countries, and so the coinfection rate is very high in such countries.^19^, ^20^

Understanding the coinfection rate has an input to design effective prevention and control intervention strategies to reduce the dual effect of morbidity and mortality. However, there are few studies reported in similar settings in Ethiopia. Thus, the aim of this study was to explore the intestinal parasitic coinfection rate and its risk factors in selected health facilities at Hawassa city, Ethiopia.

## 2 MATERIALS AND METHODS

### 2.1 Study design and settings

A facility‐based cross‐sectional study was conducted from September to December 2020 in Hawassa city. Hawassa city is located 273 km far from the capital city of Ethiopia, Addis Ababa. The city administration covers 157.2 sq. kms and has eight subcities. Lake Hawassa is also found in this city. The total population of the city was 359, 358 according to the 2007 Ethiopian census report.^21^

There are two government hospitals and 10 health centers in the city. All these health institutions provide healthcare services, including TB clinics, which provide TB diagnosis and treatment for patients coming from the catchment areas. Two hospitals (Hawassa Comprehensive Specialized Referral Hospital and Adare General Hospital) and three health centers (Millennium Health Center, Tiltie Health Center, and Tabor Health Center) were selected and included. Study participants were recruited proportionally among the five TB clinics based on their TB patient flow. A systematic sampling technique was employed for the recruitment of the study participants in each health institution based on the sampling frame (medical registration number). Confirmed pulmonary tuberculosis (PTB) patients who were on antiparasitic treatment within 2 weeks before data collection were excluded. All demographic, clinical, and laboratory data were collected and registered for each study participant.

### 2.2 Sample size determination

The sample size was determined using a single population proportion formula, according to the following assumptions: 95% confidence level, 5% margin of error, and proportion of intestinal parasitosis among PTB patients in Arba Minch, 26.3%.^17^ The initial sample size was 200. Adding a 10% nonresponse rate, the final sample size was 220 participants. All PTB patients were diagnosed and confirmed by GeneXpert and smear microscopy.

### 2.3 Data collection and quality assurance

All demographic, clinical, and predisposing factor parameters were collected using a pretested structured questionnaire. All data were collected by five trained data collectors working in each health institution. Patient charts and medical records were reviewed. The questionnaire was developed in English and translated to Amharic (local language) during data collection. All data were checked for completeness and consistency on a daily basis. A unique identification number was written for each patient for identification.

### 2.4 Stool sample collection, processing, and examination

Each study participant was instructed on how to collect and then transport the collected fresh stool samples to the respective laboratory sections. Approximately 3 g of fresh stool specimens were collected using a labeled, dry, clean, leak‐proof, and wide‐mouthed 5 mL stool cup with an applicator stick. The collected specimens were transported and processed immediately in the parasitology laboratory.

#### 2.4.1 Direct saline microscopy

A portion of the fresh stool sample was processed using the saline wet mount technique and examined using direct Olympus microscope. One or two drops of normal saline were placed at the center of one slide and was mixed with 50 mg stool. A uniform suspension was made and covered with a cover slip. The smear was examined using ×10 and ×40 objective lenses and ×10 eyepiece power to examine protozoa trophozoites and cysts, and helminths ova and larvae.^22^

#### 2.4.2 Formal‐ether concentration method

The remaining stool samples were preserved using 10% formalin and transported using a cooled box (kept at 2–8°C) to the parasitology laboratory. An estimated pea‐size of stool sample was emulsified in 4 mL of 10% formol water and mixed very well. Four milliliters of diethyl ether was added after sieving the emulsified stool and centrifuged at 750 –1000*g* for 1 min. IPs were sedimented at the bottom of the tube. Smears were prepared from the sediment and examined in the same way as the direct saline method for the presence or absence of IPs.^22^

#### 2.4.3 Laboratory quality control

Positive and negative stool samples were processed for quality assurance of the test procedure and the ability to observe and detect parasites under a microscope. Two senior laboratory experts were observed the microscopic results, and if there was a discordance between them, the third expert confirmed the final result. All quality control measures were undertaken.

### 2.5 Data analysis

Data were entered and then coded using Epi‐Data software version 3.2 and exported to SPSS software version 22 for analysis. Model fitness was checked using the Hosmer–Lemeshow test. Descriptive statistics and logistic regression analysis were employed. Bivariate crude odds ratio and multivariate‐adjusted odds ratio (AOR) logistic regression analyses were carried out to identify risk factors associated with dual infections. Variables with a *p* < 0.25 during bivariate analysis were entered into the multivariate logistic regression analysis. A *p*‐value less than 0.05 was considered statistically significant.

## 3 RESULTS

### 3.1 Demographic and clinical characteristics of study participants

A total of 214 study participants were enrolled in this study. The majority of the study participants were males 119 (55.6%), rural dwellers 120 (56.1%), and protestant in religion 149 (69.6%). The ages of the study participants ranged from 41 to 65 years old. More than half of the study participants earned less than 2000 Ethiopian birr ($53.99 USD) per month (Table 1).

**Table 1:** Demographic and clinical characteristics of PTB patients attending five health institutions at Hawassa city from September to December 2020 (*N* = 214).

Characteristics	Frequency (*n*, %)
Sex	
Male	119 (55.6)
Female	95 (44.4)
Age in years	
<18	4 (1.9)
18–40	62 (29)
41–65	129 (60.3)
>65	19 (8.9)
Residence	
Urban	94 (43.9)
Rural	120 (56.1)
Education	
Able to read and write	111 (51.9)
Primary school	41 (19.2)
High school	28 (13)
University/college	34 (15.9)
Occupational status	
Government employee	37 (17.3)
Farmer	61 (28.5)
Merchant	24 (11.2)
Daily laborer	21 (9.8)
Student	40 (18.7)
Housewife	31 (14.5)
Religion	
Protestant	149 (69.6)
Orthodox	36 (16.8)
Muslim	13 (6.1)
Catholic	9 (4.2)
Others	7 (3.3)
Monthly income (Ethiopian birr)	
≤2000	127 (59.3)
>2000	87 (40.7)
Marital status	
Married	163 (76.2)
Single	34 (15.9)
Divorce	14 (6.5)
Widow	3 (1.4)
Duration of anti‐TB treatment in months	
≤2	129 (60.3)
>2	85 (39.7)
Presence of underlying chronic diseases	
Yes	73 (34.1)
No	141 (65.9)
Wash raw fruits and vegetables before eating	
Yes	80 (37.4)
No	134 (62.6)

Abbreviations: PTB, pulmonary tuberculosis; TB, tuberculosis.

### 3.2 Magnitude of IPs

**Figure 1:**
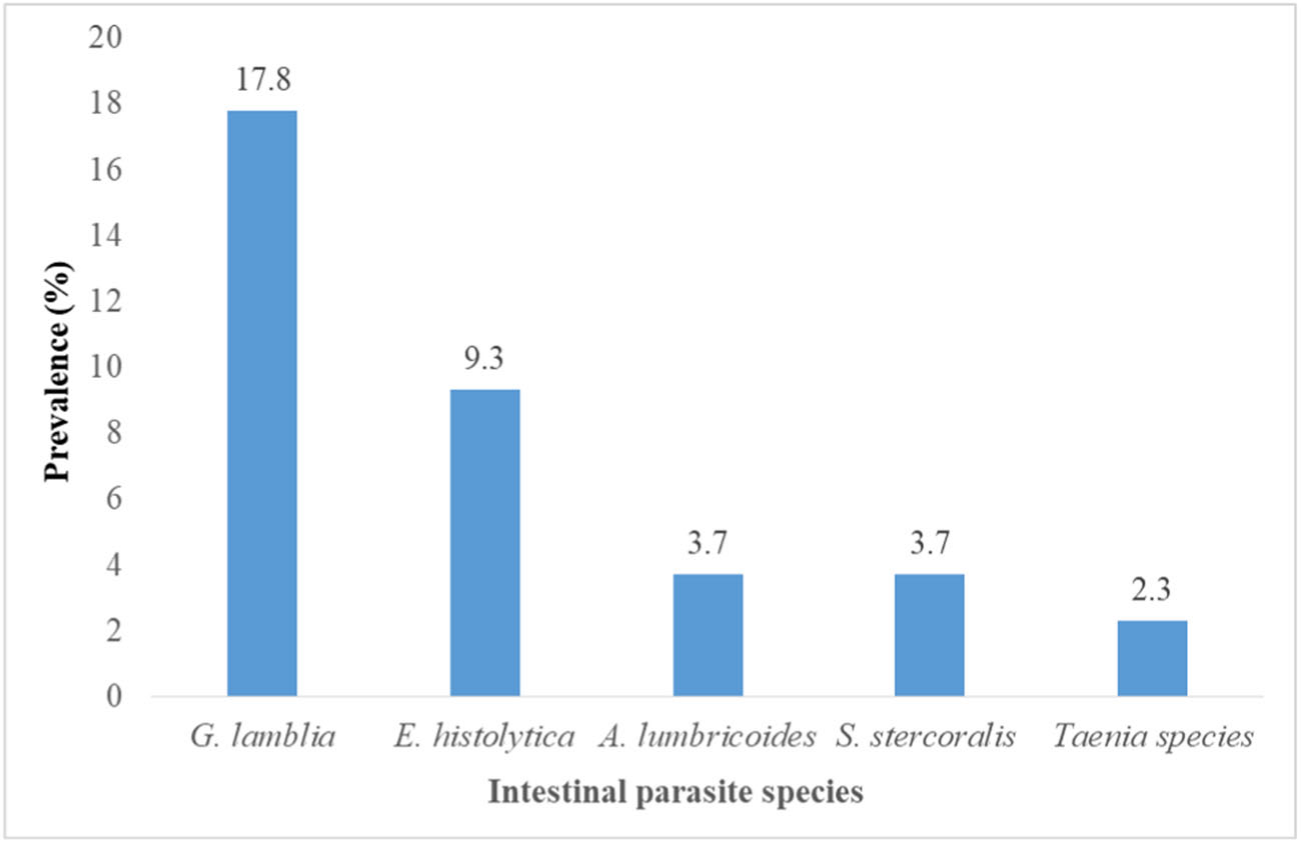
Prevalence of IPs‐MTB coinfection in patients attending at five health institutions in Hawassa city from September to December 2020. IP, intestinal parasite; MTB, *Mycobacterium tuberculosis*.

The overall rate of IPs‐MTB coinfection was 36.9% (79/214). The prevalence of intestinal protozoa was 27.1% and intestinal helminths were 9.8%. The predominant parasitic coinfection was *Giardia lamblia* 38 (17.8%), followed by *Entamoeba histolytica* 20 (9.3%), and the least was *Taenia* species (2.3%) (Figure 1).

### 3.3 Associated risk factors for acquisition of IPs‐MTB coinfection

Bivariate analysis showed that sex, age, marital status, religion, occupation, and education level of patients were all not statistically significant predictors for intestinal parasitosis and had more than a 0.25 *p*‐value. Those risk factors with *p*‐values  < 0.25 became candidates for multivariate analysis and were considered to have a crude association (Table 2).

**Table 2:** Risk factors associated with IPs‐MTB coinfection at five health institutions in Hawassa city from September to December 2020.

Variables	Category	IP coinfection		COR (95% CI)	*p*‐Value	AOR (95% CI)	*p*‐Value
		Yes	No				
Residence	Rural	38	82	1.67 (1–2.92)	0.07	2.42 (1.226–4.8)	0.01
	Urban	41	53	Ref		Ref	
Monthly income (ETB)	≤2000	51	73	1.68 (0.94–2.98)	0.08		
	>2000	28	62	Ref			
Duration of anti‐TB treatment	≤2 months	57	72	2.3 (1.25–4.12)	0.007	3.1 (1.5–6.3)	0.002
	>2 months	22	63	Ref		Ref	
Presence of underlying chronic disease	Yes	43	30	4.8 (2.6–8.8)	0.001	6.9 (3.4–14)	<0.001
	No	36	105	Ref		Ref	
Wash raw fruits and vegetables before eating	Yes	14	66	Ref		Ref	
	No	65	69	3 (1.6–5.84)	0.001	4.14 (1.91–9)	<0.001

Abbreviations: AOR, adjusted odds ratio; CI, confidence interval; COR, crude odds ratio; IP, intestinal parasite; MTB, *Mycobacterium tuberculosis*.

We found that intestinal parasitosis coinfection had a statistically significant association with risk factors such as patients being rural dwellers, not washing fruits and vegetables before eating, the presence of chronic diseases, and taking anti‐TB treatment in the intensive phase. PTB patients who were living in rural areas were 2.42 times (AOR = 2.42, 95% confidence interval [CI]: 1.23–4.8) more likely to develop intestinal parasitosis than their counterparts. Those patients who did not wash fruits and vegetables before eating were 4.14 times (AOR = 4.14, 95% CI: 1.91–9) more likely to develop intestinal parasitosis than those who washed fruits and vegetables before eating. Patients who were in the early phase of anti‐TB treatment were 3.1 times (AOR = 3.1, 95% CI: 1.5–6.5) more likely to develop an intestinal parasitic infection (IPI) than those who were at the continuous phase of anti‐TB treatment. Those patients who had underlying chronic diseases were 6.9 times (AOR = 6.9, 95% CI: 3.4–14) more likely to develop intestinal parasitosis than those who had no chronic diseases (Table 2).

## 4 DISCUSSION

TB is a major public health problem that is frequently associated with parasitic diseases. The coexistence of parasitic diseases and TB is more common in resource‐limited countries due to lack of or low preventive and control measures for the parasitic diseases.

Our findings showed that the prevalence of IP‐MTB coinfection was 36.9% (95% CI: 30.3–43.4). This finding was higher than studies reported in different countries, such as China (7.3%),^23^ Brazil (19.6%),^11^ Southern Ethiopia (26.3%),^17^ and Northwest Ethiopia (2%).^24^ This might be due to variations in population, infection prevention and control practices, and level of awareness of IP transmission. Moreover, this variation is also due to differences in the parasite detection methods; for example, studies reported in China and Brazil used in‐vitro culture, modified Kato‐Katz detection tools, and others, which are relatively more sensitive and specific. Our findings also showed comparable results to studies conducted in Northern Ethiopia (33.3%, 36.8%, and 40.5%).^2^, ^13^, ^25^

Moreover, one study in Northwest Ethiopia (71%) reported a higher rate of helminth infection than the present study.^3^ This variation might be due to differences in laboratory protocol followed, the number of study participants, and improvement of the health extension programs in our study area. In addition, the number of stool samples collected and examined may lower our study result since three stool samples were collected and examined by previous study, but our study collected and examined only a single stool sample for analyses. School‐based deworming of soil‐transmitted helminths and schistosomiasis is being practiced annually in Ethiopia, which might reduce the transmission and prevalence of IPs in our study. Currently, Ethiopia has over 40,000 health extension workers who were trained and are working in the community to prevent infectious disease transmission and basic curative services.^26^ The priority goals of the health extension programs in Ethiopia are to create awareness on how to construct and utilize latrines, avoid open defecation, and advocate good personal and environmental hygiene practices within the community to decrease the rate of IPIs and other diseases.

Our findings revealed that a significant number of intestinal parasitic species were coinfected with PTB patients. *G. lamblia* causes the highest coinfection rate (17.8%), followed by *E. histolytica* (9.3%), *Ascaris lumbricoides* (3.7%), *Strongyloides stercoralis* (3.7%), and the least *Taenia* species (2.7%). The rate of IPs‐MTB coinfection varies according to various studies. *A. lumbricoides* is the most prevalent parasite reported in previous studies.^2^, ^17^, ^27^ On the reverse, other studies showed a high prevalence of hookworms in China (4.3%), and Northwest Ethiopia (11.1%), and *S. stercoralis* in Brazil (72%).^13^, ^23^, ^28^ This variation in the distribution of IP species might be due to differences in geographical location, poor personal hygiene, types of detection methods such as Lutz, Kato‐Katz, and Baerman‐Moraes methods used by a study in Brazil, study settings, and environmental factors. Previous studies also documented that walking in barefoot was associated with helminth infection.^13^, ^23^ This study revealed that the majority of the study participants were rural residents, and shoes‐wearing habits are low.

In the present study, we have shown risk factors associated with IPs‐MTB coinfection. IPIs and TB can be risk factors for each other.^3^ One study revealed that patients infected with TB harbor more IPIs than TB‐free household members,^16^ suggesting that people with IPIs are more susceptible to getting TB.

The multivariate analysis indicated that rural dwellers, eating raw fruits and vegetables without washing, the presence of chronic diseases, and patients in the early phase of anti‐TB treatment were risk factors for IP‐MTB coinfection. Rural dwellers were 2.42 times more likely to develop IPIs than urban dwellers. Alemayehu et al.^13^ also documented similar findings. Rural communities are less likely to change their lifestyle including thier low level of shoe‐wearing habits, low socioeconomic status, and poor personal hygiene, which would increase the risk of being coinfected by IPs and MTB.

Persons who eat raw fruits and vegetables without washing were 4.14 times more likely to develop intestinal parasitic coinfections than their counterparts. Parasite ova or cysts are also ingested via contaminated fruits and vegetables, unwashed hands, and contaminated food or water to initiate the parasitic infection. It is not only due to eating without washing but also because consumption of raw fruits and vegetables is potentially high risk for the acquisition of parasitic infections. Those TB patients with underlying chronic diseases were 6.9 times more likely to be infected by IPs compared to their counterparts. Chronic diseases decrease the host immune response as a result of the infection process and the drug or chemotherapy given to the patient, which favors the IPs‐MTB coinfections.

We found a statistically significant association between anti‐TB treatment duration and intestinal parasitic coinfection. Those TB patients who were on the early anti‐TB treatment (intensive phase) were three times more likely to develop IPI than those who were on the continuous phase. This might be due to that the load of the TB bacilli is high in the early phase of anti‐TB treatment (intensive phase) which lasts for 2 months and reflects lower immunity and becomes more susceptible for parasitic coinfection compared to the late phase of anti‐TB treatment. Moreover, as the duration of the anti‐TB treatment continues, the patient immunity is becoming better and stronger, and the probability to be infected by IPs is becomes lower. On the contrary, previous studies indicated that anti‐TB treatment and its duration had no significant association with intestinal parasitosis coinfection.^17^, ^23^, ^28^

## 5 CONCLUSIONS

The rate of IPs‐MTB coinfection was 36.9% in this study. The predominant parasitic coinfection was *G. lamblia*, followed by *E. histolytica*. Being rural dwellers, eating fruits and vegetables without washing, presence of underlying chronic diseases, and being at the early stage of anti‐TB treatment were statistically associated with the acquisition of intestinal parasitic coinfection.

We recommended that healthcare providers should screen TB patients for intestinal parasitic coinfection, and provide continuous health education on personal hygiene practices, and infection prevention and control practices to ensure good prognosis and treatment outcomes of TB patients.

## AUTHOR CONTRIBUTIONS

**Agumas Shibabaw**: Conceptualization; data curation; formal analysis; investigation; methodology; project administration; writing—original draft. **Mihret Tilahun**: Data curation; writing—review and editing. **Alemu Gedefie**: Data curation; writing—review and editing. **Zenawork Sahle**: Writing—review and editing. **Melaku A. Belete**: Writing—review and editing. **Hussen Ebrahim**: Writing—review and editing. **Habtu Debash**: Writing—review and editing. **Bekele Sharew**: Writing—review and editing.

## CONFLICT OF INTEREST STATEMENT

The authors declare no conflict of interest.

## ETHICS STATEMENT

The study was conducted in accordance with the Declaration of Helsinki, and approved by the Research and Ethics Review Committee of the College of Medicine and Health Sciences (Reference number: CMHS‐211/12/20, date of approval: August 02, 2020), Wollo University. Permission letter was obtained from each study site before the commencement of data collection. Written informed consent was obtained from all participants involved in this study. All the information collected from each study participant were handled confidentially by omitting their names. Positive results were reported to physicians for further treatment and care.

## TRANSPARENCY STATEMENT

The lead author Agumas Shibabaw affirms that this manuscript is an honest, accurate, and transparent account of the study being reported; that no important aspects of the study have been omitted; and that any discrepancies from the study as planned (and, if relevant, registered) have been explained.

## Data Availability

The raw data supporting the results and conclusions of this article will be made available without restriction by the corresponding author.
